# Acute Venous Disease Anomalies in Critically Ill COVID-19 Patients

**DOI:** 10.7759/cureus.27067

**Published:** 2022-07-20

**Authors:** Adriana Torres-Machorro, Claudia Lerma, Rodrigo Lozano-Corona, Flavio Adrian Grimaldo-Gómez

**Affiliations:** 1 Department of Vascular Surgery, National Institute of Cardiology, Mexico City, MEX; 2 Department of Electromechanical Instrumentation, National Institute of Cardiology, Mexico City, MEX; 3 Department of Hematology, National Institute of Cardiology, Mexico City, MEX

**Keywords:** deep vein thrombosis, covid-19, critically ill covid-19 patients, risk of covid-19 mortality, superficial vein phlebitis, deep vein thrombosis (dvt), prognostic score, elevated d-dimer, venous anomalies

## Abstract

Introduction: Other entities besides deep vein thrombosis (DVT) affecting the venous system, such as superficial vein phlebitis (SVP) and superficial vein thrombophlebitis (SVT), receive poor attention in the literature. However, both entities may propagate proximally into the deep venous system and progress to a DVT. To our knowledge, the relevance of other venous findings such as SVP or SVT in coronavirus disease 2019 (COVID-19) patients has not been evaluated. This work aimed to assess the clinical, biochemical, and hematological variables associated with the incidence of acute venous diseases, such as DVT, SVP, and SVT, in a cohort of 74 critically ill COVID-19 patients and their association with mortality.

Methods: Given the high thrombotic risk, all patients underwent venous imaging with bedside ultrasound. Clinical variables were obtained from medical records. Comparisons were made by the chi-square test or Fisher’s exact test. We constructed Kaplan-Meier curves and used Cox proportional hazard models to calculate hazard ratios for dichotomized risk factors to identify predictors of mortality. SPSS version 21.0 (IBM Corp., Armonk, NY) was used for statistical analysis.

Results: SVP occurred in 28 patients (37.8%), DVT in 22 patients (29.7%), and 28 patients died (37.8%). Elevated D-dimer was associated with DVT but not with SVP. Neither SVP nor DVT was associated with mortality. After adjusting for age, elevated troponins (OR: 2.4, 95% CI: 1.1-5.4), platelets < 244 cell/mm^3^ (2.4, 1.1-5.6), and IMPROVE (International Medical Prevention Registry on Venous Thromboembolism) bleeding score > 7 (2.8, 1.3-6.3) were predictors of mortality.

Conclusions: Acute venous findings such as SVP and DVT are highly prevalent and independent of mortality in critically ill COVID-19 patients. These entities are not related, although they may occur synchronically. DVT is frequently presented as an asymptomatic distal bilateral finding associated with elevated D-dimer, decreased ferritin, and higher vasoactive drug use but independent from chronic venous disease. Interestingly, elevated troponins, decreased platelets, and a prognostic value > 7 of the IMPROVE bleeding score were predictors of mortality in this group of critically ill COVID-19 patients.

## Introduction

Infection due to type 2 coronavirus may lead to acute respiratory syndrome. It affects several vascular beds and is associated with coagulopathy. The latter is favored by venous stasis, activation of coagulation, and vascular dysfunction/injury [1] of endothelial cells [2].

Markers such as D-dimer, high levels of fibrinogen [3], factor VIII, and von Willebrand factor [4] have a primary role in diagnosing and mortality prognosis of different venous thromboembolism (VTE) disease phenotypes, including asymptomatic deep vein thrombosis (DVT) in coronavirus disease 2019 (COVID-19) [3,5]. VTE occurs predominantly in hospitalized and critically ill COVID-19 patients with high-dose thromboprophylaxis or despite full-dose anticoagulation therapy [5,6]. In this context, the overall VTE prevalence is about 30% [3,5], thus suggesting a relatively high prevalence compared to other infections in the intensive care unit (ICU) setting [7].

Other entities besides DVT affecting the venous system, such as superficial vein phlebitis (SVP) and superficial vein thrombophlebitis (SVT), receive poor attention in the literature. However, both entities may propagate proximally into the deep venous system and progress to a DVT. This situation prompts a more aggressive treatment, especially when affecting great extensions (more than 5 cm in length) or when located at less than 1 cm from the saphenofemoral or saphenopopliteal junction [8]. To our knowledge, the relevance of other venous findings such as SVP or SVT in COVID-19 patients has not been evaluated.

This work aimed to assess the clinical, biochemical, and hematological variables associated with the incidence of acute venous diseases such as DVT, SVP, and SVT in a cohort of critically ill COVID-19 patients. Also, we investigated the effect of these acute venous system pathologies on mortality.

## Materials and methods

Subjects

We analyzed 74 patients admitted consecutively to the ICU, diagnosed with severe COVID-19 pneumonia due to their high thrombotic risk and the elevated Padua score, which is intended to evaluate the estimated risk of VTE in hospitalized patients. Patients included were diagnosed with COVID-19 infection by real-time polymerase chain reaction and provided written informed consent.

Study protocol

The study was conducted according to the National Institute of Cardiology “Ignacio Chávez” Research Ethics Committee guidelines (CONBIOETICA-09-CEI-014-20160708) and is in compliance with the principles outlined in the Helsinki Declaration.

Demographics, clinical history, blood workup, and treatment protocols were obtained from the medical records. Patients underwent bilateral calf circumference measurement at 10 cm below the tibial tuberosity (considering a significant difference of more than 3 cm) and a complete deep and superficial vein Doppler ultrasound including the great saphenous vein (GSV), small saphenous vein (SSV), and their junctions with the deep venous system. Also, calf, popliteal, femoral, and iliac veins (assessed by phasicity and augmentation responses) were studied.

SVP was defined as an inflammatory disorder in the wall of the GSV or the SSV observed as a compressible vein with a double or thickened wall in Doppler ultrasound and a patent monophasic Doppler flow [9]. SVT was defined whenever we found the previous imaging findings without compression and absent Doppler flow, compatible with thrombus [10]. DVT was defined as a non-compressible deep venous segment with or without increased venous diameter, echogenic thrombus within the lumen, or spectral color Doppler signal absence. Bedside ultrasound was performed with a high-resolution mode B imaging system (SonoScape X5 Digital Color Doppler Ultrasound System (SonoScape Medical Corp., Shenzhen, China) equipped with an L741 frequency linear probe: 4.0-16.9 MHz). All patients were screened for indirect signs of pulmonary embolism by a 3P-A probe (frequency: 1.0-6.0, sweep sector: 90º).

Because of the increased thrombotic risk associated with COVID-19, patients with Padua score > 4 were treated with full anticoagulation doses per institutional protocol. Those with lower Padua scores received high thromboprophylactic doses. The anticoagulation scheme was instaurated either with low-molecular-weight heparin (enoxaparin) or unfractionated heparin, according to the glomerular filtration rate from day one. A multidisciplinary consensus was created for decision-making in prescribing anticoagulation in high IMPROVE (International Medical Prevention Registry on Venous Thromboembolism) bleeding score patients. Anticoagulation adjustments were performed for those with DVT and full-dose anticoagulation by titrating anti-factor-Xa activity to upper limit levels. On the other hand, those with augmented platelet activity were treated with acetylsalicylic acid in addition to full-dose anticoagulation.

Therapeutic anticoagulation treatment was continued until death and extended anticoagulation was given whenever they were released from the ICU for at least 15 days with no documented DVT. Otherwise, they were treated for at least three months or until the D-dimer was normal.

Due to the pandemic outbreak, patients were reassessed by bedside Doppler ultrasound whenever D-dimer had new drastic elevations during their ICU stay, according to the daily measurement results.

Statistical analysis

The nominal variables are described as an absolute value (percentage) and were compared between groups by the chi-square test or Fisher’s exact test. Kolmogorov-Smirnov tests were applied to continuous variables to test for normal distribution, which is described as mean ± standard deviation or median (25-75 percentile), and were compared by Student’s t-test or Mann-Whitney U test, accordingly. The variables with significant differences (p < 0.05) between groups were selected for a logistic regression analysis to assess the association of such variables with SVP or DVT through estimation of the odds ratio (95% CI).

We compared all study variables among those who died during the follow-up and those who survived. To consider the effect of time on the predictive values of the patient’s characteristics, we performed a survival analysis that involved the time to event (e.g., days from admission). The time to develop the event (e.g., mortality) was displayed by constructing Kaplan-Meier curves, and the significance was estimated by a log-rank test, which compares the survival distributions between groups defined by the patient’s characteristics. Cox proportional hazard models were used to calculate hazard ratios for dichotomized risk factors to identify predictors of mortality. Cutoff values to dichotomize the risk factors were calculated with receiver operating characteristics (ROC) curve analysis, with the best cutoff value selected from the point of the ROC curve with the shorter orthogonal distance to the optimum cutoff value (0.1). The statistical analysis was performed with SPSS version 21.0 (IBM Corp., Armonk, NY).

## Results

SVP occurred in 28 patients (37.8%), DVT in 22 patients (29.7%), and a total of 28 patients died (37.8%). Of the patients, 17% had previous heart disease/intervention. Only two had emphysema or asthma, three had previous VTE (one DVT + pulmonary embolism and two isolated DVTs), and two had a unilateral saphenectomy. The history of SVT was not included in the previous VTE history. Only two patients had previous malignant disease records, which correspond to the early stages of endometrial and breast cancer. Both were treated long before the severe COVID-19 pneumonia hospitalization and were considered cured. No other patient had active known cancer or malignancy. As only one patient presented with a 7-cm length SVT, we analyzed this patient in the SVP group.

Some of the patients had manifestations of chronic venous diseases as stated in the clinical signs of the CEAP (Clinical-Etiology-Anatomy-Pathophysiology) classification [11] that were not associated with the acute venous findings. In our cohort, one patient presented with C5, six patients with C4a, two with C3, two with C2, five with C1, and 58 patients with C0. Subgroup analysis showed that the majority of our subjects had no chronic signs of venous disease. The DVT group had two C1, one C3, and three C4a. The SVP group had four C1, one C2, and four C4a.

SVP had the following distribution: 39.2% involved both GSV and SSV bilaterally, 39.2% affected one of the superficial veins, 10.7% involved both SSV, and a similar percentage involved both GSV. Patients showed no signs suggestive of SVT. Table 1 shows the study variables compared according to the occurrence of SVP. Patients who developed SVP had higher creatinine compared to those who did not develop SVP. All other study variables were similar between groups, including the Padua score (p = 0.873). None of the SVP patients required reassessment due to abrupt D-dimer increases.

**Table 1:** Study variables grouped by the occurrence of superficial vein phlebitis (SVP). CRP = C-reactive protein; LDH = lactate dehydrogenase; PT= prothrombin time; ICU = intensive care unit; IMPROVE = International Medical Prevention Registry on Venous Thromboembolism.

	SVP		P-value
Variables	Yes (N = 28)	No (N = 46)	
Age (years)	61 ± 11	56 ± 13	0.146
Sex (male)	24 (85.7%)	31 (67.4%)	0.103
Body mass index (kg/m^2^)	28.9 ± 5.2	29.3 ± 5.0	0.752
Obesity	11 (39.3%)	21 (45.7%)	0.592
Diabetes	13 (46.4%)	12 (26.1%)	0.073
Hypertension	12 (42.9%)	14 (30.4%)	0.278
Total comorbidities	1 (1-3)	1 (1-2)	0.209
Smoking habit	11 (39.3%)	14 (30.4%)	0.435
Lymphocytes (cell/μL)	60 (450-900)	650 (500-1000)	0.324
Thrombocytes (x10^3^/μL)	253.89 ± 124.89	277.2 ± 105.46	0.393
Creatinine (mg/dL)	1.1 (0.8-1.8)	0.7 (0.5-1.3)	0.016
Ferritin (ng/mL)	1081.00 ± 569.21	996.87 ± 728.84	0.605
CRP (mg/L)	292.25 ± 122.84	239.29 ± 122.76	0.760
LDH (U/L)	426 (353-589)	368 (286-443)	0.153
D-dimer (μg/mL)	0.562 (0.300-0.915)	0.478 (0.240-0.900)	0.668
Maximum D-dimer (μg/mL)	1.700 (0.710-3.400)	0.980 (0.560-2.400)	0.150
PT (sec)	12 (12-13)	12 (12-13)	0.720
Fibrinogen (g/L)	6.28 ± 1.89	5.49 ± 1.79	0.770
Troponins (pg/mL)	15 (6-46)	23 (8-115)	0.218
Days with heparin use	6 (3-11)	3 (2-8)	0.105
High dose prophylaxis (days)	0 (0-2)	1 (0-3)	0.151
Full dose anticoagulation (days)	4 (2-9)	2 (0-5)	0.058
Vasoactive drugs	17 (60.7%)	24 (52.2%)	0.473
COVID-19 ICU stay	4 (2-12)	3 (2-6)	0.303
Hospital stay (day)	20 (10-43)	17 (12-28)	0.717
Padua	6 (5-6)	6 (5-6)	0.873
IMPROVE bleeding	6 (5-7)	6 (5-7)	0.957

Only one patient presented with proximal DVT, as the majority of thrombus was located in the gastrocnemius plexus. Patients showed no significant difference in calf perimeter between legs, thus they were diagnosed with subclinical DVT. Within the DVT group, 50% had a bilateral distal DVT presentation, except for the patient who presented with proximal right DVT and distal left DVT. DVT patients showed no abrupt increases in D-dimer during their ICU stay. The study variables grouped by DVT occurrence are shown in Table 2. Compared to those who did not develop DVT, patients with DVT had lower ferritin, higher D-dimer, and maximum D-dimer, and a more significant proportion of patients required vasoactive drugs. There was no significant difference in the other variables, including the Padua score (p = 0.056). Only the proximal DVT patient underwent a spiral CT that ruled out a pulmonary embolism.

**Table 2:** Study variables grouped by the occurrence of deep vein thrombosis (DVT). CRP = C-reactive protein; LDH = lactate dehydrogenase; PT = prothrombin time; ICU = intensive care unit; IMPROVE = International Medical Prevention Registry on Venous Thromboembolism.

	DVT				P-value				
Variable	Yes (N = 22)	No (N = 52)							
Age (years)	62 ± 14	56 ± 12		0.092					
Sex (male)	17 (77%)	38 (73%)		0.779					
Body mass index (kg/m^2^)	30.6 ± 5.8	28.7 ± 4.8		0.142					
Obesity	12 (54.5%)	20 (38.5%)		0.202					
Diabetes	6 (27%)	19 (37%)		0.592					
Hypertension	11 (50%)	15 (29%)		0.081					
Total comorbidities	2 (1-2)	1 (1-3)		0.321					
Smoking habit	7 (32%)	18 (35%)		1.000					
Lymphocytes (cell/μL)	600 (500-800)	600 (500-1000)		0.584					
Thrombocytes (x10^3^/μL)	259 ± 80	272 ± 125		0.660					
Creatinine (mg/dL)	0.9 (0.7-1.8)	0.8 (0.6-1.4)		0.229					
Ferritin (ng/mL)	816 ± 424	1121 ± 735		0.029					
CRP (mg/L)	276 ± 111	252 ± 130		0.447					
LDH (U/L)	407 (286-474)	393 (304-561)		0.805					
D-dimer (μg/mL)	1.07 (0.50-5.8)	0.45 (0.25-0.63)		0.001					
Maximum D-dimer	4.15 (1.8-6.8)	0.73 (0.55-1.60)		<0.001					
PT (sec)	13 (12-14)	12 (11-13)		0.113					
Fibrinogen (g/L)	6 ± 2	6 ± 2		0.757					
Troponins (pg/mL)	44 (6-76)	16 (8-53)		0.590					
Heparin use (days)	3 (2-8)	4 (2-9)		0.774					
High dose prophylaxis (days)	0 (0-3)	0 (0-2)		0.814					
Full dose anticoagulation (days)	3 (2-8)	2 (1-6)		0.500					
Vasoactive drugs	17 (77.3%)	24 (46.2%)		0.021					
Padua score	6 (5-6)	5 (5-6)		0.056					
IMPROVE bleeding score	7 (5-7)	6 (5-7)		0.051					
COVID-19 ICU stay	3 (2-8)	3 (2-7)		0.837					
Hospital stay (day)	16 (10-22)	18 (12-38)		0.232					

Globally, patients were treated with therapeutic anticoagulation in 80% and with a high thromboprophylaxis dose in 20%. Full-dose anticoagulation was prescribed to 86% of the patients who died and 75% of the survivors.

There was no significant association between SVP and DVT, mortality and SVP, and mortality and DVT (p > 0.05, chi-squared tests). Only 10 patients presented with concomitant DVT and SVP. From this group, six patients presented with bilateral DVT, which would correspond in every case with the ipsilateral SVP affected limb. The rest of the patients with concomitant DVT and SVP presented with SVP in the ipsilateral and contralateral limbs in three patients, and in one case, DVT and SVP affected only the right limb.

Table 3 shows the study variables grouped by mortality. Contrasting with survivors, patients who died were older, had less body mass index (BMI), had more proportion of diabetics, and had lower lymphocytes and thrombocytes, higher creatinine, and higher D-dimer. They also had more patients with a three- or six-fold increase in D-dimer (DD 3x and DD 6x, respectively), elevated troponins, more significant IMPROVE bleeding score, and more patients who required invasive mechanical ventilation (IVM). Notably, the Padua score, aimed to identify thrombosis risk, was not different between patients who survived and those who died. Cutoff values to dichotomize continuous variables from this list were estimated using the ROC curve analysis in Table 4.

**Table 3:** Study variables grouped by the occurrence of mortality. CRP = C-reactive protein; AKI = acute kidney injury; LDH = lactate dehydrogenase; DD = D-dimer; DD 3x = three-fold increase in D-dimer; DD 6x = six-fold increase in D-dimer; PT = prothrombin time; DVT = deep vein thrombosis; SVP = superficial vein phlebitis; IMPROVE = International Medical Prevention Registry on Venous Thromboembolism; IVM = invasive mechanical ventilation.

	Mortality		P-value
Variable	Yes (N = 29)	No (N = 45)	
Age (years)	63 ± 11	54 ± 12	0.003
Sex (male)	22 (75.9%)	33 (73.3%)	1.000
Body mass index (kg/m^2^)	27.6 ± 4.25	30.3 ± 5.4	0.029
Obesity	10 (34.5%)	22 (48.9%)	0.222
Diabetes	15 (51.7%)	10 (22.2%)	0.009
Hypertension	10 (34.5%)	16 (35.6%)	0.925
Total comorbidities	1 (1-2)	1 (1-2)	0.365
Smoking habit	11(37.9%)	14 (31.1%)	0.545
Lymphocytes (cell/μL)	500 (500-700)	800 (500-1000)	0.025
Lymphopenia (cell/μL)	25 (86.2%)	30 (66.7%)	0.101
Thrombocytes (x10^3^/μL)	228.68 ± 86.09	294.53 ± 121.71	0.015
Thrombocytopenia	6 (20.7%)	3 (6.7%)	0.072
Creatinine (mg/dL)	1.4 (0.8-2.3)	0.8 (0.5-1.2)	0.022
AKI on admission	10 (34.5%)	22 (50%)	0.191
Ferritin (ng/mL)	1142.25 ± 777.84	968.73 ± 593.17	0.288
CRP (mg/L)	287.96 ± 121.77	244.83 ± 124.25	0.151
CRP drastic rise	12 (41.4%)	17 (37.8%)	0.757
LDH (U/L)	376 (286-581)	395 (332-476)	0.782
D-dimer (μg/mL)	0.600 (0.440-1.690)	0.460 (0.240-0.740)	0.026
Maximum D-dimer (μg/mL)	1.690 (0.960-2.400)	0.900 (0.540-3.100)	0.065
DD drastic rise (μg/mL)	15 (51.7%)	22 (48.9%)	0.812
DD 3x (μg/mL)	22 (75.9%)	23 (52.3%)	0.043
DD 6x (μg/mL)	18 (62.1%)	14 (31.8%)	0.011
PT (sec)	13 (12-13)	12 (11-13)	0.065
Fibrinogen (g/L)	5.86 ± 2.08	5.78 ± 1.74	0.864
Troponins (pg/mL)	39 (10-60)	14 (6-65)	0.232
Elevated troponins	16 (55.2%)	14 (31.1%)	0.040
Heparin use (days)	4 (2-7)	3 (2-10)	0.899
High dose prophylaxis (days)	0 (0-2)	0 (0-3)	0.199
Full dose anticoagulation (days)	4 (1-7)	2 (1-6)	0.799
Full dose anticoagulation	25 (86.2%)	33 (75%)	0.376
DVT global	10 (34.5%)	12 (26.7%)	0.473
SVP global	14 (48.3%)	14 (31.1%)	0.137
Padua	6 (5-6)	6 (5-6)	0.551
IMPROVE bleeding	7 (5-7)	6 (4-7)	0.005
Hospital stay (day)	14 (10-25)	20 (12-44)	0.142
COVID-19 ICU stay	4 (2-7)	3 (2-7)	0.481
IVM	23 (79.3%)	25 (56.8%)	0.048
Vasoactive drugs	20 (69%)	21 (46.7%)	0.093

**Table 4:** Receiver operator characteristic (ROC) curve analysis to identify the best cut-off for selected variances to predict mortality. AUC = area under the curve; 95% CI = 95% confidence interval.

Variable	Sensitivity	Specificity	AUC (95% Ci)	P-value
Age ≥ 62 years	62%	67%	0.70 (0.58-0.82)	0.004
BMI > 27.9 Kg/m^2^	59%	64%	0.65 (0.52-0.78)	0.034
Lymphocytes < 750 (cell/μL)	79%	53%	0.65 (0.53-0.78)	0.026
Platelets < 244 (cell/mm^3^)	69%	64%	0.67 (0.54-0.79)	0.017
Creatinine > 1.25 (mg/dL)	55%	79%	0.69 (0.54-0.84)	0.022
D-dimer > 0.57 (μg/mL)	62%	64%	0.65 (0.53-0.78)	0.026

**Figure 1:**
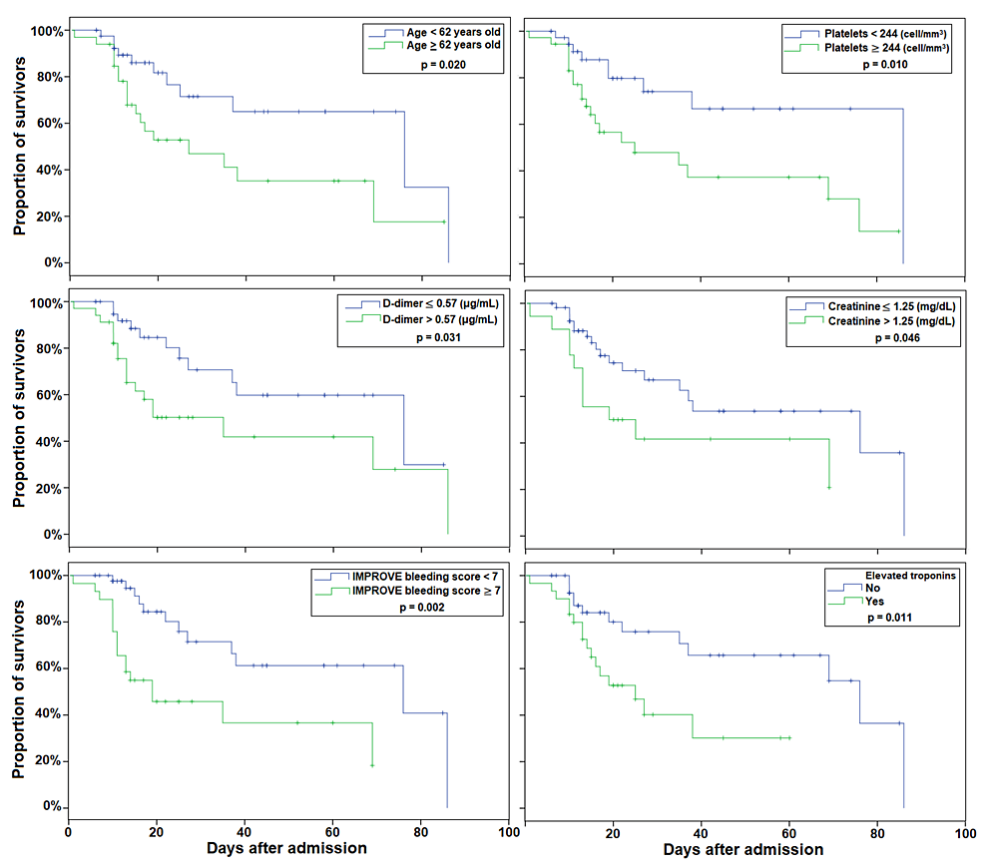
Survival analysis with Kaplan-Meier curves in critically ill COVID-19 patients. IMPROVE = International Medical Prevention Registry on Venous Thromboembolism.

**Figure 2:**
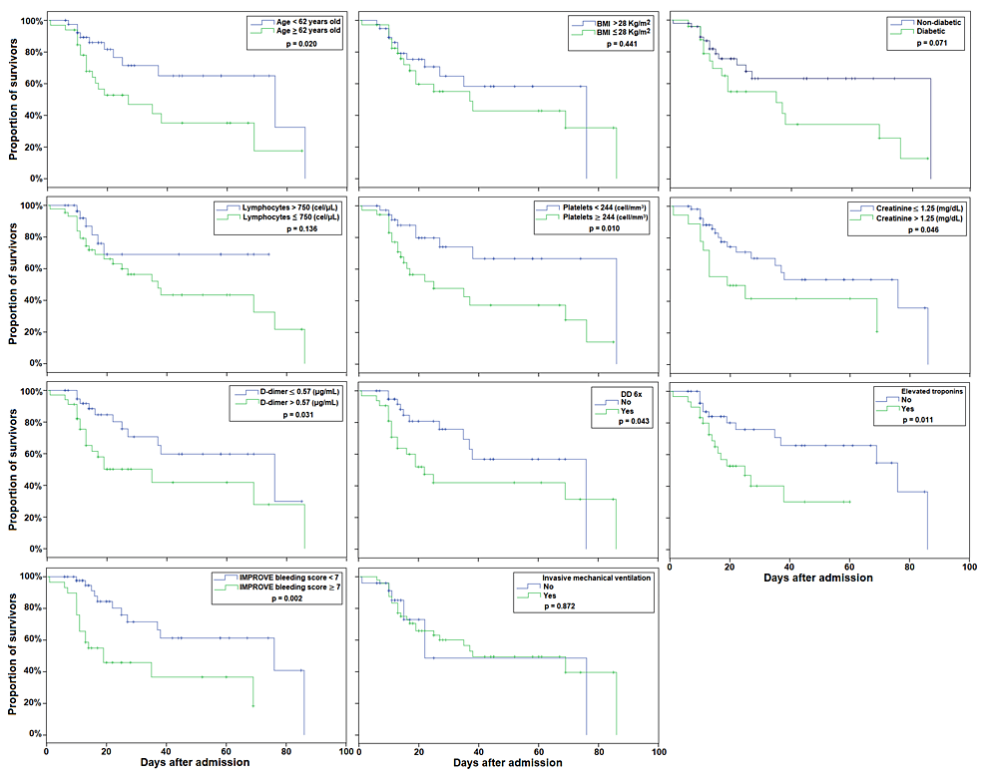
Kaplan-Meier curves of other tested variables with no significant predictive value for mortality in critically ill COVID-19 patients. DD 6x = six-fold increase in D-dimer; IMPROVE = International Medical Prevention Registry on Venous Thromboembolism.

The survival analysis with Kaplan-Meier curves is shown in Figure 1. The following characteristics were significant predictors of mortality in these critically ill COVID-19 patients: age ≥ 62 years old, platelets < 244 cell/mm^3^, creatinine > 1.25 mg/dL, D-dimer > 0.57 μg/mL, elevated troponins, and IMPROVE bleeding score ≥ 7. The Kaplan-Meier curves of other tested variables with no significant predictive value for mortality are shown in Figure 2.

Cox regression analysis (Table 5) showed that age > 62 years, elevated troponins, platelets < 244 cell/mm^3^, D-dimer > 0.57 μg/mL, and IMPROVE bleeding > 7 were factors associated with higher risk for mortality. After adjusting by age > 62 years, elevated troponins, platelets < 244 cell/mm^3^, and IMPROVE bleeding > 7 remained significantly associated.

**Table 5:** Cox regression analysis of variables associated with mortality. HR = hazard ratio; 95% CI = 95% confidence interval; DD 6x = six-fold increase in D-dimer; IMPROVE = International Medical Prevention Registry on Venous Thromboembolism.

	Univariate		Adjusted by age > 62 years	
Variable	HR (95% CI)	P-value	HR (95% CI)	P-value
Age > 62 years	2.4 (1.1-5.3)	0.025	-	-
Creatinine > 1.25 (mg/dL)	2.1 (0.9-4.6)	0.054	-	-
Elevated troponins	2.7 (1.2-5.9)	0.015	2.4 (1.1-5.4)	0.033
Platelets < 244 (cell/mm^3^)	2.8 (1.2-6.4)	0.014	2.4 (1.1-5.6)	0.039
D-dimer > 0.57 (μg/mL)	2.2 (1.0-4.8)	0.038	2.1 (0.9-4.6)	0.068
DD 6X	2.2 (1.0-4.6)	0.050	-	-
IMPROVE bleeding > 7	3.3 (1.5-7.2)	0.003	2.8 (1.3-6.3)	0.011

## Discussion

Many vascular conditions, such as endothelialitis, vascular endothelial injury, and coagulation dysfunction, have been described in some COVID-19 patients and are likely associated with disease severity [12]. In our study, many patients had a chronic venous disease that was not associated with the acute venous findings. The SVP finding was higher than DVT incidence in critically ill COVID-19 patients, but it was not statistically associated with DVT, probably suggesting an independent clinical entity. D-dimer values were elevated in every patient, but there was no significant difference in the D-dimer of those with or without SVP. This may be explained by the sample size and also because they were not associated with thrombus as DVT did. Also, it may suggest that the ultrasonographic finding was located in the endothelial wall due to the SARS-CoV-2 endothelial tropism described in the literature [12]. Notably, SVP affected either or both GSV or SSV and was independent of vasoactive drug use. Thus, this may be another manifestation of severe COVID-19 infection due to its proclivity to affect the endothelial cells [12] independent of the vasospasm favored by vasoactive drugs. There is still uncertainty about the evolution and significance of this abnormal ultrasonographic finding. Hence, identifying potential markers of increased risk for SVP remains an open question.

Regarding DVT, patients were asymptomatic and had prominent bilateral infrapopliteal affection according to other earlier reports [5,13]. Our series reported a higher incidence of bilateral involvement of distal DVT (50%), contrasting with a 4.5% reported by Demelo-Rodriguez et al., but similar to that reported by Le Jeune et al. [13,14]. Also, DVT prevalence was elevated despite high-dose thromboprophylaxis or full-dose anticoagulation [5,15]. Patients who developed DVT had elevated D-dimer and decreased ferritin, confirming previous descriptions [5,16,17]. Other factors previously related to DVT, including age, obesity, and smoking habits, were not confirmed in our population.

DVT patients in our study required a higher proportion of vasoactive drug usage, which indirectly denotes the severe inflammatory effect of the disease on hemodynamic stability. Considering that vasoactive agents produce microcirculatory changes on target organs for the production of microthrombi during disseminated intravascular coagulation (DIC) [18], the possible role of vasoactive drugs in the development of DVT in critically ill COVID-19 patients without DIC is still pending. Nevertheless, other studies have reported atypical DVT risk factors in COVID-19 patients, including hypoalbuminemia, higher Sequential Organ Failure Assessment (SOFA) score, and elevated D-dimer [16], highlighting the relevance of vasoactive drugs included in the SOFA score [19]. Moreover, the SOFA score has also recently proved its utility for predicting DVT in this group of patients [20].

DVT or SVP did not impact mortality in critically ill COVID-19 patients, which is congruent with other publications. On the contrary, age, diabetes, elevated troponins, elevated D-dimer, and elevated creatinine were associated with mortality, comparable to other reports [21].

Hematologic markers such as decreased lymphocyte and platelet count were also confirmed to be associated with mortality in our study [22]. Increased lactate dehydrogenase and other inflammatory markers like ferritin and C-reactive protein, previously described as mortality-associated factors, were not found to be significant in this group of patients [22]. Differences in our population may reflect its ethnic diversity, and the impact of this factor remains unclear.

Obesity is one of the most prevalent comorbidities affecting almost 40% of the Latin-American population [23]. Along with being overweight, both factors are well-known pro-inflammatory states that contribute to the COVID-19 cytokine storm. Even though obesity is a known poor prognosis predictor in COVID-19 [24], in our study, the positive association corresponded to those who had overweight or normal weight. Similarly, Wu et al. did not find significant participation of obesity in COVID-19 disease severity after multivariate analysis [25]. Furthermore, paradoxical protective effects of obesity in severe sepsis and septic shock cases have been previously reported for other diseases [26]. Variations may also be related to the nutritional status [27], environmental factors, and the complexity of genetic differences absent in other continental groups [28] that may play a role in these protective associations.

Even though the Padua score is an assessment model for identifying patients at risk of VTE, in our study, this score was not different in the groups compared by SVP, DVT, or mortality. Contrary to the IMPROVE bleeding score that may be related to mortality as a result of the many factors included in this score shown to be significantly associated with mortality [22]. IMPROVE bleeding and SOFA’s prognostic value utility is clinically relevant and must be underlined [21].

According to our analysis, critically ill COVID-19 patients would be at an increased mortality risk when aged ≥ 62 years old, have a BMI > 27.9 kg/m^2^, lymphocytes < 750 cell/μL, platelets < 244 cell/mm^3^, creatinine > 1.25 mg/dl, and D-dimer > 0.57 μg/mL. This risk profile derives from quantitative analysis to obtain an objective cutoff value that increases mortality and may vary between samples and populations. Moreover, survival analysis and Cox regression models confirmed that the variables associated with increased mortality risk are age > 62 years, creatinine < 1.25 mg/dl, elevated troponins, platelets < 244 cell/mm^3^, D-dimer > 0.57 μg/ml, a six-fold increase in D-dimer, and IMPROVE bleeding score > 7. Being persistently significant are the elevated troponins and IMPROVE bleeding score, and platelets < 244 cell/mm^3^ after age-adjusted analysis.

The applicability of our results may be limited due to our small sample size and follow-up, even though this is one of the few publications in our population related to mortality and the first reporting SVP. Increasing the sample size in future works would aid in developing further prognostic models to predict mortality, outcomes, and thrombotic complications. Results may be restricted to our population due to genetic, epigenetic, and environmental factors exclusively interacting with the virus in our region. These factors should be noted when interpreting our results.

## Conclusions

Acute venous findings such as SVP and DVT are highly prevalent and independent of mortality in critically ill COVID-19 patients. These entities are not related, although they may occur synchronically. DVT is frequently presented as an asymptomatic distal bilateral finding associated with elevated D-dimer, decreased ferritin, and higher vasoactive drug use but independent from chronic venous disease. Interestingly, elevated troponins, decreased platelets, and a prognostic value > 7 of the IMPROVE bleeding score were predictors of mortality in this group of critically ill COVID-19 patients.

## References

[1] Esmon CT (2009). Basic mechanisms and pathogenesis of venous thrombosis. Blood Rev.

[2] Hoffmann M, Kleine-Weber H, Schroeder S (2020). SARS-CoV-2 cell entry depends on ACE2 and TMPRSS2 and is blocked by a clinically proven protease inhibitor. Cell.

[3] Eljilany I, Elzouki AN (2020). D-dimer, fibrinogen, and IL-6 in COVID-19 patients with suspected venous thromboembolism: a narrative review. Vasc Health Risk Manag.

[4] Escher R, Breakey N, Lämmle B (2020). ADAMTS13 activity, von Willebrand factor, factor VIII and D-dimers in COVID-19 inpatients. Thromb Res.

[5] Torres-Machorro A, Anguiano-Álvarez VM, Grimaldo-Gómez FA (2020). Asymptomatic deep vein thrombosis in critically ill COVID-19 patients despite therapeutic levels of anti-Xa activity. Thromb Res.

[6] Klok FA, Kruip MJ, van der Meer NJ (2020). Confirmation of the high cumulative incidence of thrombotic complications in critically ill ICU patients with COVID-19: an updated analysis. Thromb Res.

[7] Boddi M, Peris A (2017). Deep vein thrombosis in intensive care. Adv Exp Med Biol.

[8] Quéré I, Leizorovicz A, Galanaud JP (2012). Superficial venous thrombosis and compression ultrasound imaging. J Vasc Surg.

[9] Decousus H, Quéré I, Presles E (2010). Superficial venous thrombosis and venous thromboembolism: a large, prospective epidemiologic study. Ann Intern Med.

[10] Di Nisio M, Wichers IM, Middeldorp S (2018). Treatment for superficial thrombophlebitis of the leg. Cochrane Database Syst Rev.

[11] Lurie F, Passman M, Meisner M (2020). The 2020 update of the CEAP classification system and reporting standards. J Vasc Surg Venous Lymphat Disord.

[12] Ackermann M, Verleden SE, Kuehnel M (2020). Pulmonary vascular endothelialitis, thrombosis, and angiogenesis in COVID-19. N Engl J Med.

[13] Demelo-Rodríguez P, Cervilla-Muñoz E, Ordieres-Ortega L (2020). Incidence of asymptomatic deep vein thrombosis in patients with COVID-19 pneumonia and elevated D-dimer levels. Thromb Res.

[14] Le Jeune S, Suhl J, Benainous R (2021). High prevalence of early asymptomatic venous thromboembolism in anticoagulated COVID-19 patients hospitalized in general wards. J Thromb Thrombolysis.

[15] Chen S, Zhang D, Zheng T, Yu Y, Jiang J (2021). DVT incidence and risk factors in critically ill patients with COVID-19. J Thromb Thrombolysis.

[16] Zermatten MG, Pantet O, Gomez F (2020). Utility of D-dimers and intermediate-dose prophylaxis for venous thromboembolism in critically ill patients with COVID-19. Thromb Res.

[17] Nelson CM, Wright GS, Silbaugh TR, Cota LJ (2009). Improving D-dimer positive predictive value for outpatients with suspected deep vein thrombosis. Perm J.

[18] Latour JG, Léger-Gauthier C, Solymoss BC (1985). Vasoactive agents and production of thrombosis during intravascular coagulation. 2-alpha-adrenergic stimulation: effects and mechanisms. Pathology.

[19] Ferreira FL, Bota DP, Bross A, Mélot C, Vincent JL (2001). Serial evaluation of the SOFA score to predict outcome in critically ill patients. JAMA.

[20] Prouse G, Ettorre L, Mongelli F (2021). SOFA score as a reliable tool to detect high risk for venous thrombosis in patients with critical stage SARS-CoV-2. Front Cardiovasc Med.

[21] Tian W, Jiang W, Yao J (2020). Predictors of mortality in hospitalized COVID-19 patients: a systematic review and meta-analysis. J Med Virol.

[22] Henry BM, de Oliveira MH, Benoit S, Plebani M, Lippi G (2020). Hematologic, biochemical and immune biomarker abnormalities associated with severe illness and mortality in coronavirus disease 2019 (COVID-19): a meta-analysis. Clin Chem Lab Med.

[23] Romero-Martínez M, Shamah-Levy T, Vielma-Orozco E, Heredia-Hernández O, Mojica-Cuevas J, Cuevas-Nasu L, Rivera-Dommarco J (2019). National Health and Nutrition Survey 2018-19: methodology and perspectives. (Article in Spanish). Salud Publica Mex.

[24] Tamara A, Tahapary DL (2020). Obesity as a predictor for a poor prognosis of COVID-19: a systematic review. Diabetes Metab Syndr.

[25] Wu J, Li W, Shi X (2020). Early antiviral treatment contributes to alleviate the severity and improve the prognosis of patients with novel coronavirus disease (COVID-19). J Intern Med.

[26] Schwarzkopf D, Fleischmann-Struzek C, Rüddel H, Reinhart K, Thomas-Rüddel DO (2018). A risk-model for hospital mortality among patients with severe sepsis or septic shock based on German national administrative claims data. PLoS One.

[27] Fedele D, De Francesco A, Riso S, Collo A (2021). Obesity, malnutrition, and trace element deficiency in the coronavirus disease (COVID-19) pandemic: an overview. Nutrition.

[28] Silva-Zolezzi I, Hidalgo-Miranda A, Estrada-Gil J (2009). Analysis of genomic diversity in Mexican Mestizo populations to develop genomic medicine in Mexico. Proc Natl Acad Sci U S A.

